# Prostate tumor OVerexpressed-1 (PTOV1) down-regulates *HES1* and *HEY1* notch targets genes and promotes prostate cancer progression

**DOI:** 10.1186/1476-4598-13-74

**Published:** 2014-03-31

**Authors:** Lide Alaña, Marta Sesé, Verónica Cánovas, Yolanda Punyal, Yolanda Fernández, Ibane Abasolo, Inés de Torres, Cristina Ruiz, Lluís Espinosa, Anna Bigas, Santiago Ramón y Cajal, Pedro L Fernández, Florenci Serras, Montserrat Corominas, Timothy M Thomson, Rosanna Paciucci

**Affiliations:** 1 Research Unit in Biomedicine and Translational Oncology, Vall d’Hebron Research Institute, Pg. Vall d’Hebrón 119-129, Barcelona 08035, Spain; 2 Functional Validation and Preclinical Research-Molecular Imaging Platform CIBBIM-Nanomedicine, Vall d’Hebron Research Institute, Barcelona 08035, Spain; 3 Networking Research Centre for Bioengineering, Biomaterials and Nanomedicine (CIBER-BBN), Instituto de Salud Carlos III, Zaragoza 50018, Spain; 4 Department of Pathology, Vall d’Hebron Hospital and Autonomous University, Barcelona 08035, Spain; 5 Cancer Research Program, Institut Municipal d'Investigacions Mèdiques and Hospital del Mar, Barcelona 08005, Spain; 6 Department of Pathology, Hospital Clínic, University of Barcelona and Institut d’Investigacions Biomèdiques August Pi i Sunyer, Barcelona, 08034, Spain; 7 Department of Genetics, Faculty of Biology and Institute of Biomedicine, University of Barcelona, Barcelona 08034, Spain; 8 Department of Cell Biology, Barcelona Molecular Biology Institute, Science Research Council, Barcelona 08034, Spain

**Keywords:** PTOV1/HES1/HEY1/Notch signaling/prostate cancer progression

## Abstract

**Background:**

PTOV1 is an adaptor protein with functions in diverse processes, including gene transcription and protein translation, whose overexpression is associated with a higher proliferation index and tumor grade in prostate cancer (PC) and other neoplasms. Here we report its interaction with the Notch pathway and its involvement in PC progression.

**Methods:**

Stable PTOV1 knockdown or overexpression were performed by lentiviral transduction. Protein interactions were analyzed by co-immunoprecipitation, pull-down and/or immunofluorescence. Endogenous gene expression was analyzed by real time RT-PCR and/or Western blotting. Exogenous promoter activities were studied by luciferase assays. Gene promoter interactions were analyzed by chromatin immunoprecipitation assays (ChIP). *In vivo* studies were performed in the *Drosophila melanogaster* wing, the SCID-Beige mouse model, and human prostate cancer tissues and metastasis. The Excel package was used for statistical analysis.

**Results:**

Knockdown of PTOV1 in prostate epithelial cells and HaCaT skin keratinocytes caused the upregulation, and overexpression of PTOV1 the downregulation, of the Notch target genes *HEY1* and *HES1*, suggesting that PTOV1 counteracts Notch signaling. Under conditions of inactive Notch signaling, endogenous PTOV1 associated with the *HEY1* and *HES1* promoters, together with components of the Notch repressor complex. Conversely, expression of active Notch1 provoked the dismissal of PTOV1 from these promoters. The antagonist role of PTOV1 on Notch activity was corroborated in the *Drosophila melanogaster* wing, where human PTOV1 exacerbated Notch deletion mutant phenotypes and suppressed the effects of constitutively active Notch. PTOV1 was required for optimal *in vitro* invasiveness and anchorage-independent growth of PC-3 cells, activities counteracted by Notch, and for their efficient growth and metastatic spread *in vivo*. In prostate tumors, the overexpression of PTOV1 was associated with decreased expression of HEY1 and HES1, and this correlation was significant in metastatic lesions.

**Conclusions:**

High levels of the adaptor protein PTOV1 counteract the transcriptional activity of Notch. Our evidences link the pro-oncogenic and pro-metastatic effects of PTOV1 in prostate cancer to its inhibitory activity on Notch signaling and are supportive of a tumor suppressor role of Notch in prostate cancer progression.

## Introduction

The PTOV1 gene and protein are expressed at increased levels in PC [1-4] and other tumors [5,6]. PTOV1 expression is detected in putative pre-neoplastic lesions of atypical adenomatous hyperplasia (AAH) [7] and its detection in pre-neoplastic high-grade prostate intraepithelial neoplasia (HGPIN) lesions from prostatic biopsies may be helpful in the early diagnosis of PC [8]. The protein consists of a tandem repeated domain, also present as a single copy in PTOV2, or MED25, a subunit of the Mediator transcriptional complex [1,9,10], conserved among higher eukaryotes, that uses novel structural modes to recruit the VP16 activation domain [11-13]. Recently, PTOV1 was shown to repress the MED25-mediated transcription of the retinoic acid (RA) receptor [14], suggesting a potential molecular mechanism underlying resistance to RA [15]. Additionally, PTOV1 may interact with the lipid-raft associated protein Flotillin-1 [16], the phosphoserine-recognizing protein 14-3-3σ [17], the BUZ/Znf-Ubp domains of the Histone deacetylase HDAC6 [18], and the ribosomal protein RACK1 [19]. Although it is difficult to ascertain how each of these interactions contributes to a possible role of dysregulated PTOV1 expression in cancer progression, this protein modulates cell proliferation, cell cycle progression [4,16], protein synthesis and gene transcription [15,19,20]. Combined these observations suggest a function for PTOV1 as an adaptor protein implicated in different cellular events and locations.

Here we report a functional interaction of PTOV1 with the Notch signaling pathway. Notch is part of an evolutionarily conserved pathway that regulates cell differentiation, proliferation and growth [21]. Following ligand binding, two subsequent proteolytic cleavages by intracellular γ-secretase release the active intracellular domain of Notch (ICN) from the cell membrane. ICN translocates to the nucleus and interacts with the CBF-1/RBP-Jκ transcription factor and directs the expression of numerous downstream target genes, including *HES1* and *HEY1*[22-25]. In the absence of ICN, CBF-1/RBP-Jκ acts as a transcriptional repressor by forming a complex that includes SMRT/NCoR, and HDAC1 [26].

In cancer, Notch signaling, initially shown to be oncogenic in human T cell acute lymphoblastic leukemia (T-ALL), and later in other tumors [27-29], was subsequently found to function also as a suppressor of tumor growth, depending on cell lineage or tissue [30-32]. In PC, several evidences suggest a tumor suppressor role of Notch signaling [33], including its action in promoting PTEN activity [34], the downregulation of Notch1 and HEY1 expression in tumors [34,35], the undetectable levels of Notch1 and ligands in PC cell lines, and the inhibition of PC cell proliferation by ICN [36]. However, additional findings, including the elevated levels of Notch ligand Jagged1 in PC and its association to recurrence, the requirement of Notch2 in the resistance to docetaxel, and the Notch1 association with aggressive PC, are suggestive of an oncogenic role [37-39].

In this work, we show that PTOV1 promotes the invasion and anchorage-independent growth of prostate cancer cells while it acts as a novel repressor of the Notch target genes *HES1* and *HEY1*. Reciprocally, a constitutively activated Notch1 receptor decreases anchorage-independent growth and invasion *in vitro. In vivo,* PTOV1 antagonizes Notch function in the *Drosophila melanogaster* wing, and it is required for full tumor growth and metastatic potentials of PC-3 prostate cancer cells in an immunodeficient mouse model. In prostate tumors, the reciprocal expression patterns observed for PTOV1 and Notch targets support our *in vitro* findings.

## Results

### PTOV1 blunts Notch transcriptional activity

The nuclear localization of PTOV1 was previously associated with higher proliferative index and tumor grade [6], suggesting a link between nuclear PTOV1 and cancer progression in different tumor types, including prostate and bladder cancers. Others have shown that, in the nucleus, PTOV1 antagonizes the transcriptional activity of complexes requiring the histone acetyl-transferase CBP [20]. Although CBP was reported to function as a classic tumor-suppressor gene in the mouse and in prostate cancer [40-43], other evidences have also suggested a role in promoting cell proliferation and prostate cancer progression [44,45]. We thus searched for interactions of PTOV1 with transcriptional networks known to participate in the progression of PC and other cancers. Notch is one such major signaling pathway, regulating the formation of the normal prostate and involved in PC [36,46,47].

**Figure 1:**
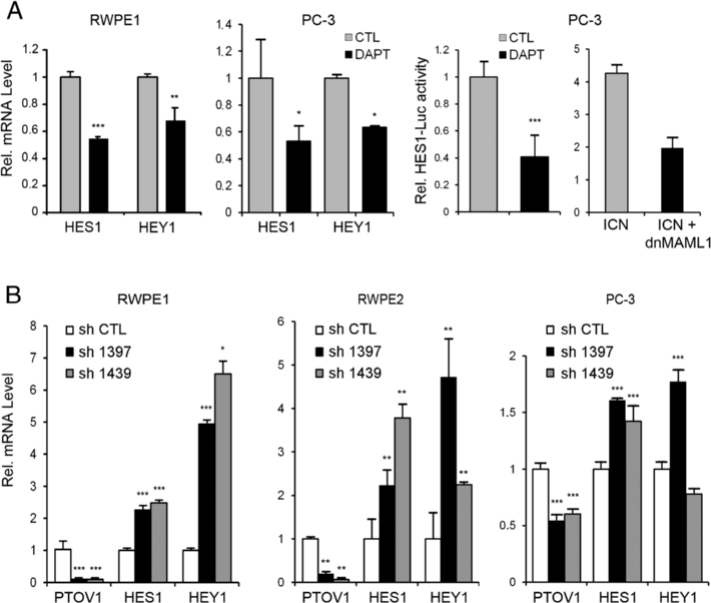
**Modulation of *HES1 *and *HEY1 *expression by Notch signaling and PTOV1 in prostate cell lines. (A)** The transcription of *HES1* and *HEY1* genes in RWPE1 and PC-3 cells is modulated by the γ-secretase inhibitor DAPT. Cells were treated with DAPT or solvent (CTL) for 4 days and *HES1* or *HEY1* transcript levels quantified by real-time RT-PCR. Right: The *HES1* promoter is modulated by DAPT and by the negative dominant-MAML1 (dnMAML1). PC-3 cells, transfected with *HES-*luciferase and *TK-Renilla*, were either treated with DAPT or co-transfected with ICN and ICN plus dnMAML1 and firefly luciferase activity, normalized to *Renilla*, determined. **(B)** Knockdown of PTOV1 in RWPE and PC-3 prostate cells by shRNAs causes the upregulation of *HES1* and *HEY1* mRNA. Cells were transduced with shRNA1397 and shRNA1439 lentiviruses and analyzed by real-time RT-PCR. Values were normalized to *RPS14* and for relative values in cells bearing control shRNA.

**Figure 2:**
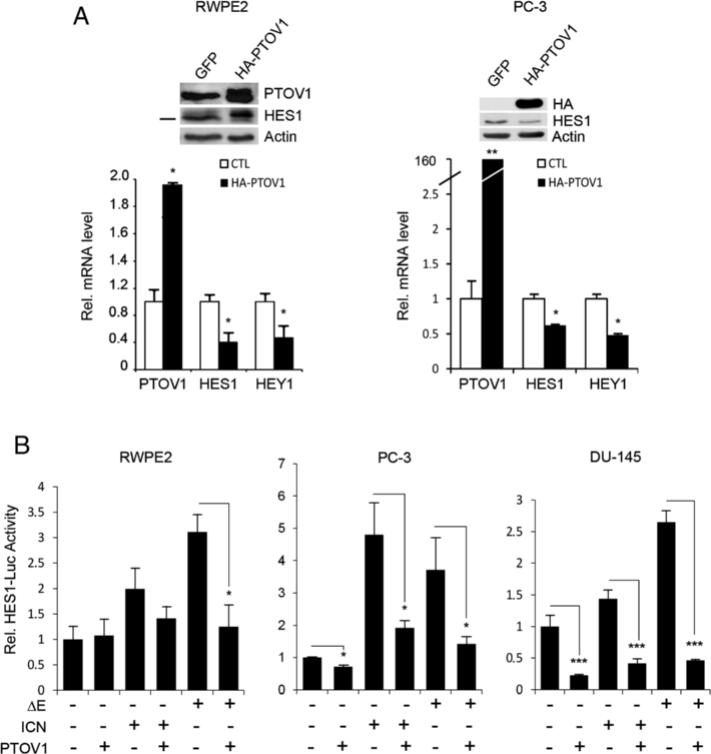
**Ectopic overexpression of PTOV1 downregulates endogenous *HEY1 *and *HES1. *(A)** Cells were transduced with lentivirus for HA-PTOV1 (black bars) or control lentivirus (white bars) and analyzed for the expression of *HES1* and *HEY1* by real-time RT-PCR. Inlet: Western blotting for the detection of ectopic HA-PTOV1 (slower migrating band in transduced cells) and endogenous HES1. **(B)** The transcriptional repressor activity of PTOV1 is downstream of Notch receptor processing. PC-3 cells, transfected with the *HES-*luciferase and *TK-Renilla* reporter plasmids, were co-transfected with ΔE (partially processed) or ICN (fully processed) forms of Notch1 without, or with HA-PTOV1, and transactivation of the *HES1*-promoter determined by luciferase assays. Statistical significance: * *p* < 0.05, ** *p* < 0.005*.*

To confirm that prostate cells have active Notch signaling [33], RWPE1 cells, derived from benign prostate epithelium, and PC-3 prostate cancer cells were treated with the γ-secretase inhibitor DAPT, known to prevent Notch processing and transcriptional signaling [48]. This treatment caused a significant downregulation of the endogenous Notch target genes *HES1* and *HEY1*, as determined by real-time RT-PCR (Figure 1A) and a comparable decline in the *HES1* promoter activity, as determined by luciferase transactivation assays (Figure 1A). A similar reduction in *HES1-*luciferase promoter activity was observed after the expression of a dominant negative form of MAML1, a transcriptional co-activator of the Notch signaling pathway [49]. Similar results were obtained with LNCaP prostate cancer cells (Additional file 1: Figure S1). Expression analysis of the four Notch receptors shows that prostate cell lines have moderate and variable levels of Notch2, Notch3 and Notch4, while Notch1 is expressed at lower levels in metastatic cell lines (Additional file 1: Figure S2). Together, these observations suggest that Notch maintains at least in part the transcription levels of *HES1* and *HEY1* genes in these cells. Next, *PTOV1* mRNA was knocked-down in prostate cells by lentiviral transduction of two distinct short hairpin RNAs (sh1397 and sh1439). These caused a significant and specific depletion of PTOV1 mRNA and protein levels in RWPE1, in ras-transformed RWPE2 cells, and in PC-3 cells (Figure 1B and Additional file 1: Figure S3) accompanied with a significant upregulation of the endogenous *HES1* and *HEY1* mRNA levels. Reciprocally, ectopic expression of HA-PTOV1 induced a significant downregulation of endogenous *HES1* and *HEY1* mRNA and protein (Figure 2A) and inhibited the transactivation of *HES1-*luciferase by ΔE or ICN, partially and fully activated forms of the Notch1 receptor, respectively, suggesting that PTOV1 acts as a repressor downstream of fully processed Notch1 (Figure 2B) in PC-3, RWPE2 and DU-145 cells. Similar Notch repressor effects by HA-PTOV1 were observed in HeLa and COS-7 fibroblasts transfected with ΔE or ICN, although not in HEK293T cells (Additional file 1: Figure S4).

### PTOV1 interacts with the Notch repressor complex at the *HEY1* and *HES1* promoters

We next analyzed whether the repressive function of PTOV1 on *HEY1* and *HES1* transcription is associated with its nuclear localization. We have previously described that PTOV1 translocation to the nucleus leads to increased cell proliferation [4,16]. In the presence of DAPT, endogenous PTOV1 and also SMRT, a component of the Notch repressor complex, showed a markedly increased nuclear localization in PC-3 and LNCaP cells (Additional file 1: Figure S5), suggesting that under conditions of inactive Notch nuclear PTOV1 and SMRT might associate with the Notch repressor complex. As indicated by pull-down assays using extracts of PC-3 cells transfected with FLAG-SMRT, PTOV1 and SMRT interacted with each other (Additional file 1: Figure S5B). Both FLAG-SMRT and endogenous SMRT proteins specifically bound the GST-A and GST-B domains of PTOV1, with the B domain showing a more efficient pull-down.

**Figure 3:**
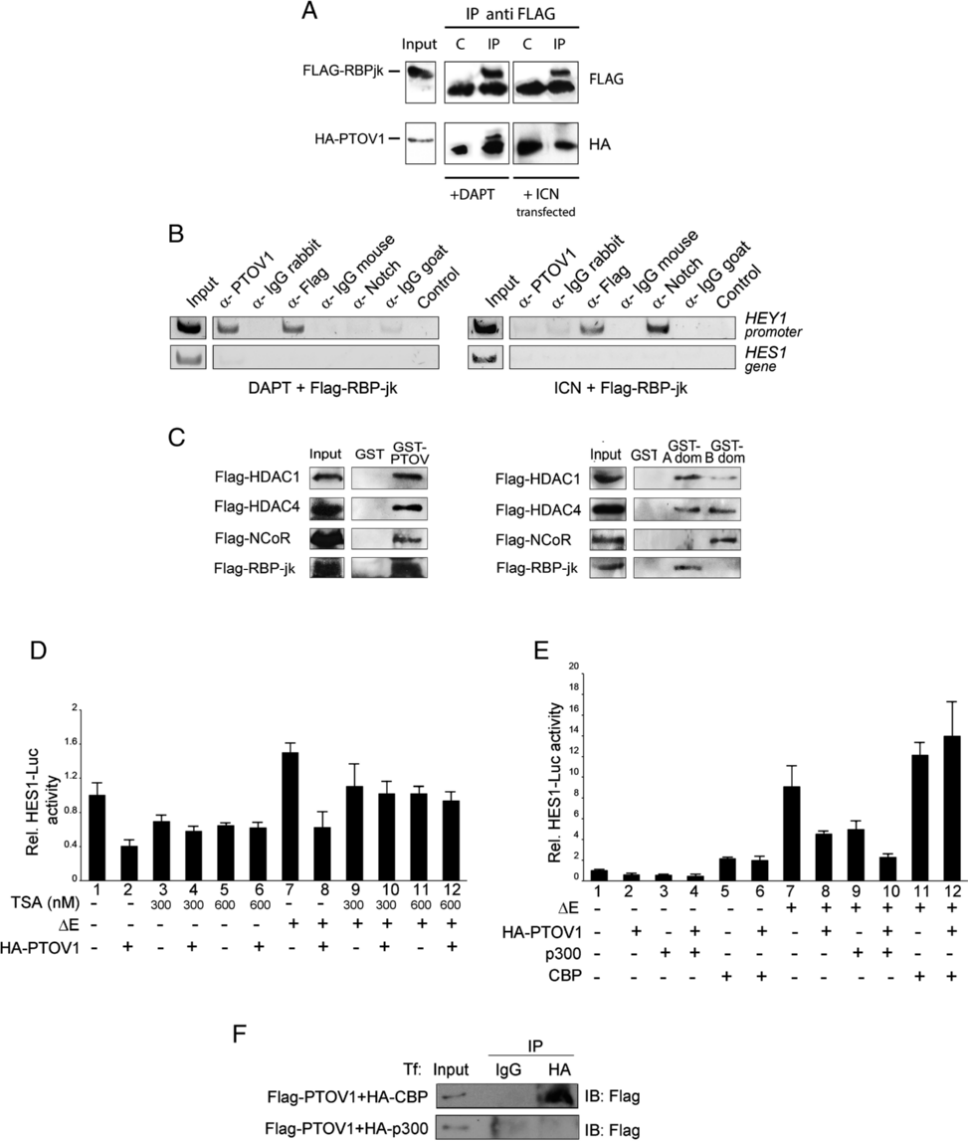
**PTOV1 interacts with RBP-Jκ and the Notch repressor complex at the *HEY1 *promoter and its repressive function requires HDACs activity and it is restrained by CBP. (A)** PTOV1 interacts with RBP-Jκ. PC-3 cells, transfected with FLAG-RBP-Jκ and HA-PTOV1, were co-transfected with ICN or treated with DAPT for 4 days. Cell lysates were immunoprecipitated with FLAG antibody or control IgG and blots were revealed with anti-HA antibody. **(B)** Endogenous PTOV1 occupies the endogenous *HEY1* promoter under conditions of Notch signaling inhibition. PC-3 cells, transfected with FLAG-RBP-Jκ, were treated with DAPT (left) or co-transfected with ICN (right). Chromatin was immunoprecipitated with antibodies to PTOV1, FLAG, Notch1, or non-specific IgGs. Associated DNA fragments were analyzed by PCR with primers specific for *HEY1* promoter regions. Primers from intragenic regions of the *HES1* gene were used as a specificity control. **(C)** PTOV1 interacts with the Notch repressor complex. FLAG-HDAC1, FLAG-HDAC4, FLAG-NCoR and FLAG-RBP-Jκ were separately transfected into PC-3 cells and tested for interaction with GST-PTOV1 (left panel) or GST-A domain, GST-B domain (right panel) or control GST beads. Bound proteins were analyzed by Western blotting with anti-FLAG. **(D)** PTOV1 repressor activity requires HDACs activity. Cells, transfected with *HES1*-luciferase*, Renilla* and the plasmids indicated, were treated with the HDAC inhibitor trichostatin A (TSA) for 48 h. Lysates were used in transactivation assays for firefly luciferase activity **(E)** Overexpression of the CBP acetyl-transferase, but not p300, overcomes the repressor activity of PTOV1 on the *HES1* promoter. Cells, transfected with p300 or CBP plus the indicated plasmids, were used in *HES1*-driven luciferase transactivation assays. **(F)** PTOV1 interacts with CBP but not with p300. Lysates from cells transfected with FLAG -PTOV1 and HA-CBP (top), or HA-p300 (bottom), were immunoprecipitated with antibody to HA, or control IgG, and analyzed by Western blotting.

The association of PTOV1 with the Notch repressor complex was confirmed by co-immunoprecipitation of PTOV1 and FLAG-RBP-Jκ (a Notch-specific DNA binding protein), observed only in the presence of DAPT but not after transfection of constitutively activated Notch (Figure 3A). To corroborate that PTOV1 interacts with the Notch-repressor complex at the *HEY1* and *HES1* promoters, we used chromatin immunoprecipitation (ChIP). When PC-3 cells were treated with DAPT, ChIP consistently revealed occupation of these promoters by endogenous PTOV1 (Figure 3B and Additional file 1: Figure S6A). RBP-Jκ, but not Notch, was also detected in these conditions. In contrast, when cells were transfected with Notch1-ICN, the *HEY1* and *HES1* promoters were occupied by ICN and RBP-Jκ, whereas PTOV1 was clearly absent. ChIP with these proteins yielded no amplified bands when using primers for internal *HES1* gene sequences and irrelevant immunoglobulins did not pull down DNA associated with these promoters. As an additional control, the co-repressor NCoR was detected at the *HEY1* promoter only in the absence of active Notch (Additional file 1: Figure S6B).

Next, the association of PTOV1 with additional elements of the Notch repressor complex was performed by pull-down experiments. In these experiments, full-length GST-PTOV1 interacted with RBP-Jκ, HDAC1, HDAC4 and NCoR (Figure 3C), whereas different components of the Notch repressor complex showed different binding preferences for either PTOV1-A domain or B domain, such that HDAC1 and HDAC4 bound to both PTOV1-A and B domains, while RBP-Jκ and NCoR showed detectable binding only to the PTOV1-A domain or the B domain, respectively (Figure 3C, right panel). These results suggest that, under conditions of inactive Notch, the nuclear localization of endogenous PTOV1 is increased and is associated with several components of the Notch repressor complex at the *HEY1* and *HES1* promoters. Activated Notch, on the other hand, provokes the dismissal of PTOV1 from these promoters.

### PTOV1 repressor activity requires active histone deacetylases

The repressive function of PTOV1 might be linked to the concurrent recruitment to these promoters of co-repressors, such as histone deacetylases (HDACs). To determine this, we treated PC-3 cells with trichostatin A (TSA), an inhibitor of HDACs [50] that relieves repression at Notch responsive promoters [51]. TSA significantly decreased the repression exerted by HA-PTOV1 on the *HES1* promoter, indicating that the PTOV1 repressive function requires active HDACs (Figure 3D). Conversely, transfection of the acetyl transferase CBP, but not p300, enhanced the transactivation of *HES1*-luciferase promoted by Notch1 and completely abolished the repressive activity of PTOV1 (Figure 3E). Consistently, PTOV1 co-immunoprecipitated with CBP (Figure 3F) [20], but not with p300. Thus, the repressive action of PTOV1 on the *HES1* promoter requires active HDACs, it is enhanced by p300 and is overcome by the expression of CBP.

### PTOV1 Suppresses notch function in *drosophila melanogaster*

**Figure 4:**
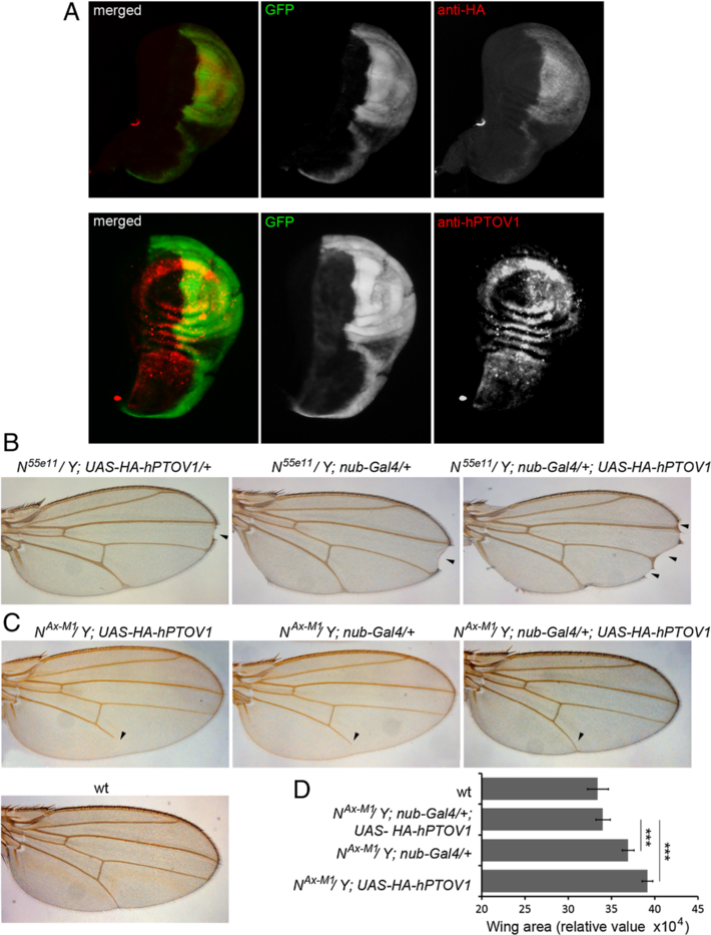
**PTOV1 antagonizes Notch activity in the *Drosophila *wing. (A)** Expression of hPTOV1 in *engrailed-Gal4UAS-GFP; UAS-HA-hPTOV1* wing imaginal discs. To confirm the activity of the transgene, the ectopic expression of hPTOV1 from the *UAS-HA-hPTOV1* was examined. The *engrailed-Gal4* line, which drives UAS-transgene expression only in the posterior compartment of the wing disc, allows to compare the levels of expression in anterior versus posterior compartments in the same tissue (imaginal disc). The expression of the transgene was observed by (1) co-activation of a UAS-GFP, (2) immuno detection of HA and (3) antibodies to PTOV1. Absence of staining in the anterior (left) half of the disc contrasts with positive staining in the posterior (right) half, demonstrating the ectopic expression of PTOV1 driven by *engrailed-Gal4*. **(B)** Overxpression of hPTOV1 exacerbates the effects of loss-of-function (LOF) of *Notch. Notch* LOF allele *N*^*55e11 *^causes a notch at the wing edge *(arrowhead*, left and middle panels) that is exacerbated by expression of hPTOV1 (*arrowheads*, right panel). **(C)** Expression of hPTOV1 suppresses the effects of *Notch* gain-of-function (GOF). The GOF *Notch* allele *N*^*Ax-M1 *^causes defects in the L5 vein (*arrowhead*, left and middle panels), suppressed by expression of hPTOV1 (*arrowhead*, right panel). A wild type (wt) wing is shown as control. **(D)** Histogram showing the quantification of the wing areas for each genotype (n > 30) (*** *p* < 0.0005).

To further corroborate the observed functional interactions between PTOV1 and the Notch pathway, we tested the effects of the expression of human PTOV1 on Notch mutant-dependent *Drosophila* wing patterns. The *Notch* mutant phenotype was first described in flies, where dosing of *Notch* produces specific patterns throughout *Drosophila* development [21,52,53]. We generated transgenic flies containing the full-length human PTOV1 cDNA tagged with HA (*hPTOV1*) under the control of the Upstream Activating Sequence (UAS) promoter to direct the expression of *hPTOV1* using the Gal4/UAS system [54]. The expression of *hPTOV1* was analyzed using the *engrailed-Gal4UAS-GFP* line that directs the expression of GFP and hPTOV1 only in the posterior part of the third instar larval wing imaginal discs (Figure 4A). To study the effect of hPTOV1 on patterns associated with loss-of-function of *Notch*, we used the *N*^*55e11*^ allele, a *Notch* null mutant that promotes notched wings [53]. When *UAS-HA-hPTOV1* was expressed in these heterozygous flies using the *nubbin-Gal4* line that drives expression in the central part of the wing disc during larval development, we observed a significant increase in the number of notches per wing (Figure 4B). The *Notch* gain-of-function phenotype (*N*^*Ax-M1*^ allele) results in failure to complete development of the most distal part of vein L5 and in a significant increase of wing size, when cultured at 25°C [53]. Expression of hPTOV1 in the *N*^*Ax-M1*^ background restored the L5 vein and the wing size to wild-type patterns, indicating suppression by hPTOV1 of the effects promoted by constitutively active Notch (Figure 4C-D). These results support the conclusion that PTOV1 acts as a negative regulator of the Notch pathway.

### PTOV1 is pro-oncogenic in prostate cancer cells

**Figure 5:**
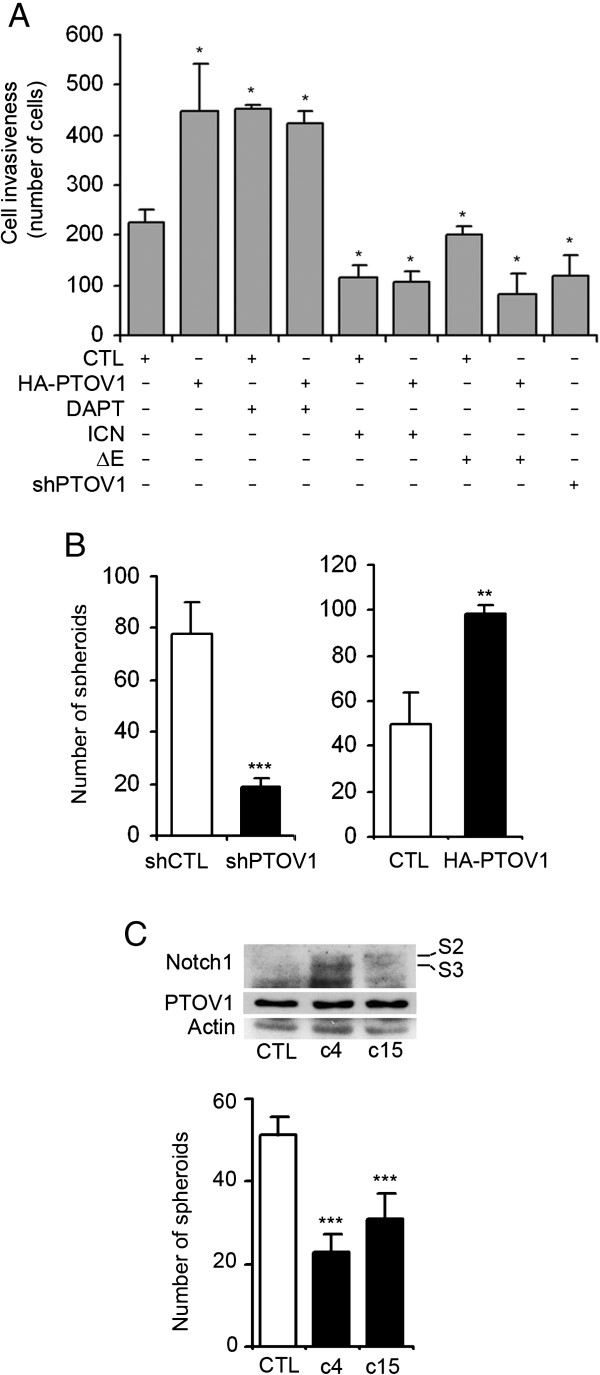
**HA-PTOV1 overexpression promotes invasion and anchorage-independent growth of PC-3 cells in contrast to the effects of exogenous Notch1 expression. (A)** PTOV1 induces *in vitro* cell invasion, in contrast to the inhibition caused by Notch1. PC-3 cells were transiently transfected with 5 μg of the indicated plasmids and/or 5μg of DNA carrier (CTL) and treated with DAPT or solvent for three days before trypsinization and plating on Matrigel-coated Transwell wells. After 24 h, invading cells were stained with Hoechst and scored. Short hairpin sh1397 was used to knockdown PTOV1 expression. Assays were performed in triplicate in at least two independent experiments. **(B)** PTOV1 promotes anchorage-independent growth of PC-3 cells. Cells stably knocked down for PTOV1 grew significantly fewer spheroids than control cells. In contrast, cells stably expressing HA-PTOV1 formed significantly more spheroids than cells transduced with control lentivirus. **(C)** Notch1 overexpression significantly decreases anchorage-independent growth of PC-3 cells. Independent clones c4 and c15, with stable expression of Notch1 as shown by Western blotting, formed significantly fewer spheroids than control cells transfected with control pcDNA3 vector. S2 and S3 indicate partial and active forms of Notch1 receptor. * *p* < 0.05, ** *p* < 0.005*,* *** *p* < 0.0005.

The expression of HA-PTOV1 in PC-3 cells significantly increased invasion compared to control cells and, reciprocally, cells expressing shPTOV1 showed that this protein is required for optimal cell invasion (Figure 5A). Importantly, the gain in invasiveness prompted by overexpression of PTOV1 was abrogated by the concomitant expression of ICN or ΔE. Similarly, knockdown of PTOV1 caused a significant reduction in the ability of PC-3 cells to from spheroids, while expression of HA-PTOV1 stimulated spheroid formation (Figure 5B). On the other hand, constitutive expression of a full-length form of Notch1 in PC-3 cells, that express low endogenous levels of this gene (Additional file 1: Figure S2), caused a significant reduction in their capacity to form spheroids (Figure 5C). These results suggest that PTOV1 promotes, and Notch signaling suppresses, key cellular properties associated with PC progression. The contrasting activities of PTOV1 and HES1 and HEY1 were also tested in HaCaT transformed skin keratinocytes, a cellular model in which Notch has known tumor suppressor functions [31,55]. In these cells, HA-PTOV1 significantly repressed *HES1* and *HEY1* expression and promoted cell proliferation and spheroid formation (Additional file 1: Figure S7). Reciprocally, knockdown of PTOV1 in HaCaT cells significantly increased the expression of these genes and decreased spheroid formation, further supporting the notion that high levels of PTOV1 suppress Notch signaling and induce oncogenic properties in different cellular contexts.

### PTOV1 is required for tumorigenesis and metastasis of PC-3 prostate cancer cells

**Figure 6:**
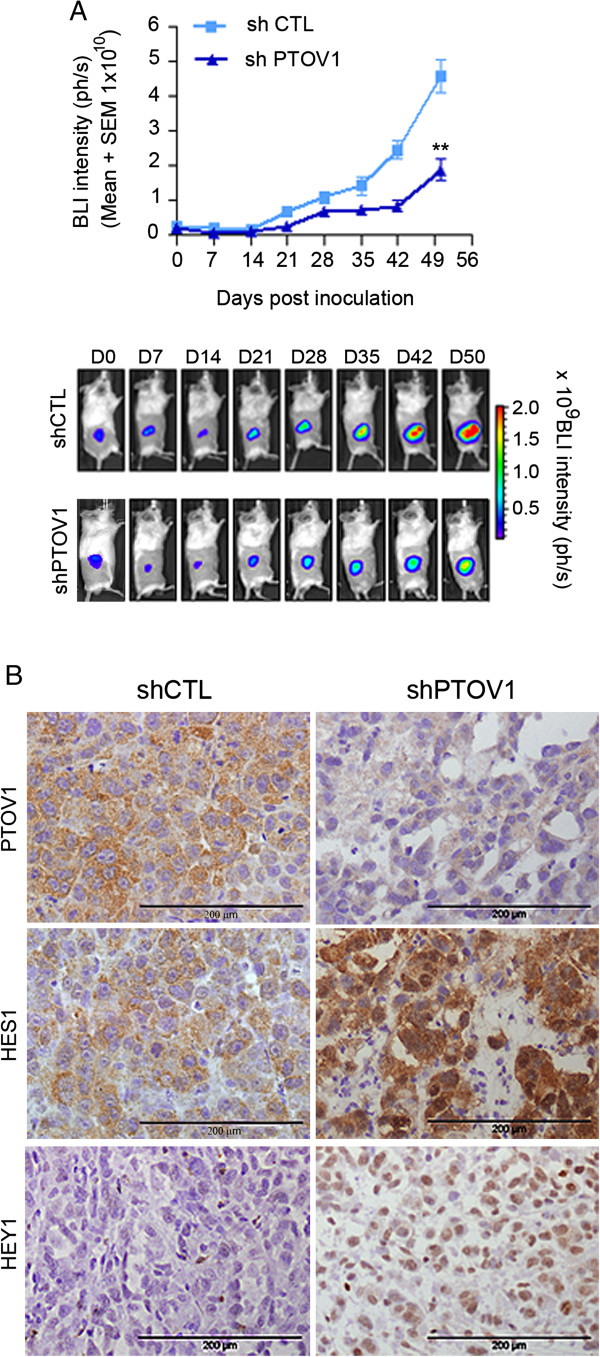
**Knockdown of PTOV1 in prostate cancer cells inhibits tumor growth and metastasis of PC-3 cells in immunodeficient mice. (A)** PTOV1 is required for optimal tumor formation of PC-3 cells *in vivo*. PC-3 cells with integrated luciferase gene (3 x10^6^), knocked down for PTOV1 (n = 5) by sh1397 or control lentivirus (n = 5), were implanted subcutaneously into the right flank of SCID-beige male mice and monitored by *in vivo* bio-luminescent imaging. Mean values + SEM are displayed. Statistically significant differences in the growth of knockdown *vs*. control cells were observed. ** *p* = 0.001. **(B)** Immunohistochemistry of explanted tumors. A strongly decreased expression of PTOV1 is detected in tumors formed by shPTOV1 cells *vs* shControl derived tumors. In contrast, tumors derived from shPTOV1 cells express high levels of HES1 and HEY1 proteins compared to shControl and grew metasases at significantly later times (*p* = 0.001) as compared to control cells.

We next tested whether PTOV1 is required for the tumorigenic and metastatic properties of PC-3 cells. Cells knocked down for PTOV1 grew significantly smaller (*p* = 0.0095) subcutaneous tumors in SCID-beige mice compared to control cells transduced with a non-targeting shRNA (Figure 6A). Immunohistochemical analysis of tumors derived from shPTOV1 cells showed strongly increased levels of HES1 and HEY1 proteins as compared to control cells (Figure 6B), consistent with a negative regulation of their expression by PTOV1. In addition, distant metastases of PTOV1 knockdown cells were detected with a significant delay (*p* = 0.001) as compared to control cells. These results provide evidence that PTOV1 is required for the expression of full tumorigenic and metastatic potentials of PC-3 cells *in vivo.*

### Reciprocal expression patterns of PTOV1 and HEY1 in prostate cancer

**Figure 7:**
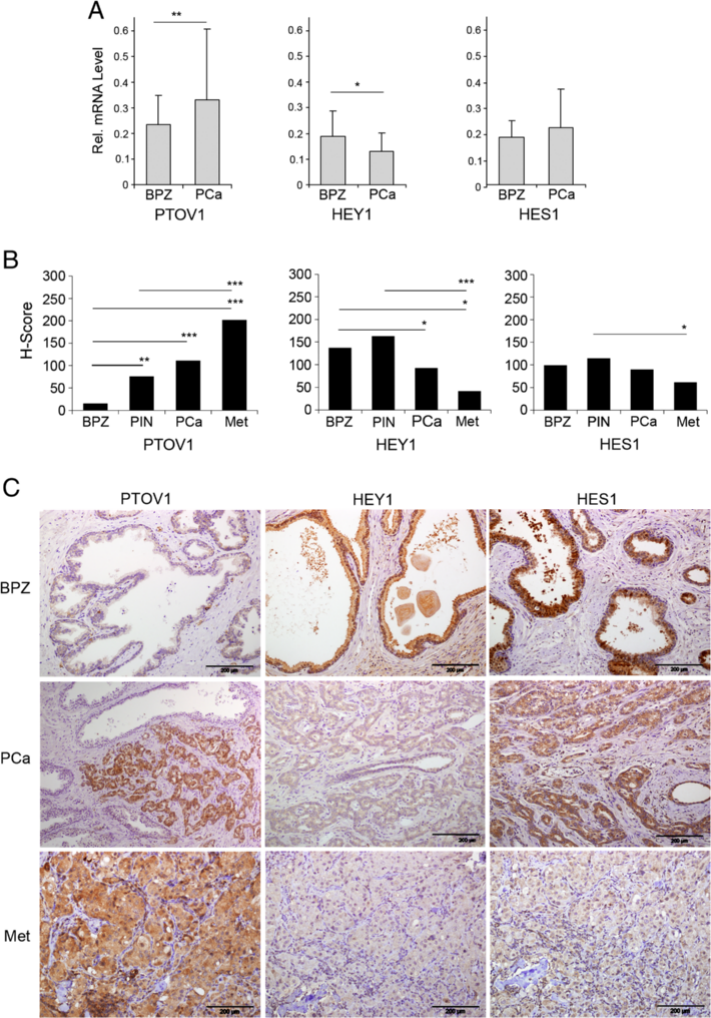
**PTOV1 displays expression patterns reciprocal to those of HEY1 and HES1 in benign prostate, primary prostate carcinoma and metastasis. (A)** PTOV1 transcripts levels increases from normal prostate to primary prostate cancer samples, while HEY1 expression declines from normal to neoplastic tissues. mRNA from 43 prostate adenocarcinomas (PCa) and their corresponding benign epithelial glands from the peripheral zone (BPZ) were analyzed by real-time PCR using specific primers and probes for *HES1*, *HEY1* or *PTOV1*. Values represent mean + SEM of mRNA expression relative to *RPS14* control. *PTOV1* levels are significantly increased in PCa compared to BPZ. *HEY1* levels in BPZ are significantly decreased in PCa. A significant inverse correlation between the expression levels of *HEY1* and *PTOV1* is evidenced (Pearson coefficient = 0.87). **(B)** The expression levels of PTOV1, as determined by immunohistochemistry, increases along with metastatic progression of PCa, while expression levels of HEY1 and HES1 declines with progression. Protein expression levels were assigned semiquantitative values by the Hscore method in benign epithelium (BPZ), pre-malignant HGPIN lesions, PCa, and 16 lymph node metastasis (Met). **(C)** Representative images from immunohistochemical staining of serial sections from PCa and metastasis (Met) with specific antibodies to HEY1, HES1 or PTOV1. Slides were counterstained with hematoxylin. PTOV1 staining is low or undetectable in BPZ, and strong expression is observed in PCa and Met. HES1 and HEY1 show strong staining in benign epithelial glands in BPZ and HGPINs. A significantly decreased staining intensity is observed for HEY1 in cancerous areas of PCa and metastasis, relative to BPZ. Staining intensity for HES1 is comparable between epithelial glands of BPZ, HGPIN and cancerous areas of PCa, but the intensity is significantly weaker in metastases (Met). Bars indicate average Hscore values.* *p* < 0.05, ** *p* < 0.005,*** *p* < 0.0005.

To know the relative contributions of PTOV1 and Notch signaling to malignancy in PC, we analyzed the expression of *PTOV1, HEY1* and *HES1* in 45 prostate adenocarcinomas and control associated benign peripheral zone (BPZ) by real-time RT-PCR. As expected [1,4], *PTOV1* expression was significantly higher in cancer with respect to BPZ (Figure 7A). In contrast, the expression levels of *HEY1* were significantly lower in tumors compared to adjacent BPZ, such that a significant inverse correlation was established between the expression levels of *HEY1* and *PTOV1* (Pearson coefficient = 0.87). The expression levels of a second Notch transcriptional target, *HES1*, were not significantly altered in tumors compared to BPZ.

Tumor tissues were analyzed at single-cell level by immunohistochemistry for the expression of PTOV1, HEY1 and HES1 proteins on serial sections from 20 primary tumors (n = 10 with Gleason < 7 and n = 10 with Gleason > 7) and 16 lymph node metastases. Epithelial cells from BPZ showed undetectable or faint staining for PTOV1, while a gradual increase in staining intensity was observed from HGPIN lesions to adenocarcinoma lesions, which generally showed a strong staining (Figure 7B-C). In metastases, the staining for PTOV1 was also significantly stronger than in BPZ. In contrast, the expression of HEY1 followed a pattern almost reciprocal to that of PTOV1 and it was significantly stronger in epithelial cells in BPZ and pre-malignant HGPIN compared to cancer and metastasis (Figure 7B-C), confirming the results at the mRNA level. HES1 expression did not show notable differences in intensity between BPZ and tumor areas, although cancerous cells showed a prevalent cytoplasmic localization (Figure 7C). Nevertheless, HES1 expression significantly decreased in metastases (Figure 7B-C), confirming a reciprocal expression pattern between PTOV1 and HES1 in metastatic lesions.

The above results bear not only on any putative roles of PTOV1 in the regulation of HES1 and HEY1 and in prostate cancer progression, but also on the controversial role of Notch in PC [33-39]. Although the results of immunohistochemical analysis show mere correlations between high PTOV1 and low HES1 and HEY1 levels, when taken in the context of the Notch repressor function for PTOV1 described above in cellular models, they are consistent with the notion that high levels of PTOV1 repress the transcriptional activity of Notch in metastatic prostate cancer.

## Discussion

A role for PTOV1 in tumor progression was suggested by previous findings showing its overexpression in PC and other neoplasms in association with increased proliferation rates and higher histological grade [1-6]. Here, we provide evidences suggesting that the pro-oncogenic function of PTOV1 is associated with a downregulation of the Notch target genes *HEY1* and *HES1*. The functional link that we have found between (i) the inhibition of Notch phenotypes in the *Drosophila* wing, (ii) the upregulation of endogenous *HES1* and *HEY1* in cells knockdown for PTOV1 and, reciprocally, their inhibition caused by ectopic expression of PTOV1 in PC cells and HaCaT keratinocytes, where Notch acts as tumor suppressor [55], and (iii) the occupancy by PTOV1 of the *HES1* and *HEY1* promoters in cells with inactive Notch receptor*,* provide strong evidences in support of the participation of PTOV1 in the regulation of Notch signaling.

PTOV1 shares similarities with SMRT, a known Notch co-repressor, in the repressive activity on *HEY1* and *HES1* promoters, the requirement for HDACs and the counteracting effects of histone acetyl transferases [56,57]. However, while SMRT is excluded from the nucleus by MEKK-1/MEK-1 or IKKα signaling [56,58], PTOV1 translocates to the nucleus upon stimulation with growth factors [4,16], and while SMRT is expressed at similar levels in BPZ and PC [59], PTOV1 is overexpressed in PC. We propose that while SMRT is generally required for the repression of Notch transcriptional activity and other signaling pathways, PTOV1 might be a facultative transcriptional co-repressor with a more restricted scope. Indeed, in response to certain mitogenic signals, PTOV1 translocates to the nucleus, where it could facilitate the transcription of genes necessary for proliferation [4,16], and invasion (Figure 5A, 5C) while simultaneously repressing Notch targets *HEY1* and *HES1* genes, as shown in the current study. Reciprocally, Notch activation excludes PTOV1 from these promoters, thus permitting the engagement of Notch-dependent programs while preventing the activation of genes that regulate general proliferation and invasion (Figure 5A, 5C). The function of PTOV1 as a Notch co-repressor could also differ from that of SKIP [60], since we show here that PTOV1 interacts with the Notch repressor complex, but not with Notch1. Similarly, SHARP, another Notch co-repressor, also interacts with the same inhibitors as PTOV1 [51,61,62], but shows different expression patterns in human tumors [6,61].

The Notch pathway is regulated by positive and negative signals [21]. Although we have shown that PTOV1 can repress the Notch-dependent expression of *HEY1* and *HES1* in the several cell lines tested, these genes are under the regulation of additional pathways in different cell types or tissues, as suggested by the observation that HES1 expression in Notch1 knockout and in CBF-1/RBP-Jκ knockout mutants is not downregulated [63,64]. Thus, although *HES1* is a *bona fide* Notch/ RBP-Jκ target, it is also regulated by different signaling cascades in tissues [65] and in fibroblasts [66,67].

The evidence presented here suggests that the recruitment of the histone acetyl transferase CBP to the *HES1* promoter overcomes the repressive action of PTOV1 on HES1 transcription. In contrast, p300, another major histone acetyl transferase, appears to enhance the transcriptional repression of HES1 by PTOV1. This suggests that these two histone acetyl transferases determine opposing transcriptional states of the *HES1* promoter, with CBP favoring a state of active transcription and p300 a state of transcriptional repression. Recent findings indicate that CBP has a stronger trans-activating function than p300 on genes whose products are negative transcription regulators, such as *HES1*[68]. This is consistent with our observations that PTOV1 and p300 cooperate to repress *HES1* transcription, while CBP relieves this repression. Of interest, p300 has been described as a positive inducer of prostate cancer progression, while CBP has been described as a tumor suppressor in the prostate [42,44,69,70]. Together with our observations that PTOV1 expression correlates positively, and HES1 expression negatively, with prostate cancer progression, these evidences may suggest that both PTOV1 and p300, which antagonize Notch target transactivation, function as positive inducers of prostate cancer progression, whereas the Notch signaling and the *HES1* activator CBP function as suppressors of prostate cancer establishment and/or progression.

Our evidences also suggest that the function of PTOV1 as a repressor of Notch signaling may have significant consequences for PC progression. Knockdown of PTOV1 in PC-3 cells led to a strong upregulation of HES1 and HEY1 both *in vitro* and in cells implanted in SCID-beige mice, accompanied with a significant delay in tumor growth and metastatic spread. These pro-oncogenic functions of PTOV1 were also observed in HaCaT keratinocytes, in which Notch behaves as a tumor suppressor [31]. In addition, our evidences suggest that high levels of PTOV1 downregulate *HES1* and *HEY1* in PC cells by promoting the recruitment of a transcription repressive complex to their promoters. This PTOV1-mediated repression requires active HDACs and is counteracted by the histone acetyl transferase CBP but not p300, suggesting that PTOV1 and Notch activities might be modulated by differential expression of these two enzymes.

In human tissues, we have found evidence of active Notch signaling in the normal prostate epithelium, as attested by the relatively high levels of expression of HES1 and HEY1, as expected [36,46], while PC metastatic samples expressed significantly lower levels of these proteins, suggestive of a Notch repressed state. PTOV1, on the other hand, showed expression patterns almost reciprocal of those for HEY1 or HES1: low levels or absent in normal epithelium and high levels in metastases. Our observations lend support to a tumor suppressor function of Notch signaling in PC, similarly to its previously demonstrated role in skin, myeloid leukemia and cervical carcinoma cells [30,55,71,72]. Additional evidences are also suggestive of a tumor suppressor function of Notch in PC, including the observations of downregulation of HEY1 and of activated Notch1 [34,35], and prevention of luminal cell differentiation and induction of proliferation in Notch1 knock-out models [35,39]. On the other hand, the activation of Notch2 detected in rare metastatic cells [38], and the overexpression of the Notch ligand Jagged-1 found in metastasis, suggest an oncogenic role for Notch in PC, although no assessment on Notch signaling was done in the same tumors [37].

## Conclusions

Taken together, our observations are compatible with a model whereby PTOV1 contributes to the initiation and progression of PC in part by counteracting the expression of *HEY1* and *HES1* genes, thus decreasing Notch signaling. These findings are also supportive of a tumor suppressor role of Notch in prostate cancer progression [33-36].

## Methods

### Cell culture, transfection and antibodies

Cell lines were obtained from the American Type Culture collection (Rockville, MD). PC-3, DU-145 and LNCaP prostate cancer cells were maintained in RPMI medium supplemented with 10% heat-inactivated FBS, 2 mM L-glutamine, 100 U/mL penicillin, 100 μg/mL streptomycin, and 0.1 mM non-essential amino acids (Life Technologies, Grand Island, NY) at 37 ºC in an atmosphere of 5% CO_2_. COS-7 fibroblasts and HaCaT keratinocytes were maintained in Dulbecco’s modified Eagle’s medium supplemented as above. Human benign prostate-derived epithelial cells RWPE1 and RWPE2 [73] were maintained in Keratinocyte-Serum-Free Medium (Invitrogen, Carlsbad, CA) supplemented with 1 μg/mL human recombinant epidermal growth factor and 10 μg/mL bovine pituitary extract. COS-7 and HeLa cells were transiently transfected for 48 h using Lipofectamine Plus reagents (Invitrogen). Prostate-derived cells were transiently transfected using the *Trans*IT^®^-Prostate Transfection Kit (Mirus Bio, Madison, WI). Cells stably transduced by HA-PTOV1 lentivirus or control were selected by flow citometry for GFP-positive cells. PC-3 cell clones c4 and c15, stably expressing Notch1, were obtained by transfection of pcDNA3-Notch1 and selection for two weeks in media containing G418. Control clones, transfected with empty vector, were also selected. DAPT (Sigma-Aldrich, St Louis, MO) was used in cell culture experiments for 4 days at 10 μM. Antibodies to SMRT, HES1 and HEY1 were from Millipore. Antibodies to Notch1 (C-20), GST (1E5) and actin (I19) were from Santa Cruz Biotechnology (Santa Cruz, CA). Antibody to γ - secretase-processed Notch1 recognizing Val 1744 was from Cell Signaling. Anti-Flag (M2), Anti-HA and anti β-tubulin were from Sigma-Aldrich.

### Plasmids

Full-length human PTOV1 cDNA was obtained from the I.M.A.G.E. Clone Consortium. Constructs harboring the *PTOV1* gene were described previously [4,16]. pIRE-LTXT vector was a generous gift of Dr. Luis Álvarez-Vallina. Partially activated Notch1 ΔE and fully activated intracellular Notch1 (ICN) constructs were kindly provided by Raphael Kopan. Full-length Notch1 in pcDNA3 (FlN1) was a kind gift of Jon Aster.

### RNA interference and lentivirus production

Short-hairpin shRNA sequences 1397 and 1439 (Sigma-Aldrich), targeting the human PTOV1 mRNA are shown in Additional file 1: Table S1. Cells transduced with lentiviral particles, were selected with 1 μg/mL puromycin (Sigma-Aldrich) for seven days.

### Transactivation assays

Cells, seeded in 12 well plates, were transfected with test plasmids plus *HES1*-*Luc,* or *HEY*-*Luc* (0.4 μg), as reporter plasmids and TK*-Renilla* (0.2 μg) as an internal control for transfection efficiency. The total amount of DNA was kept constant in each experiment by including control pCMV-HA vector. Luciferase assays were performed 48 h after transfection, following the manufacturer’s instructions (Dual Luciferase Reporter Assay Systems, Promega, Madison, WI). Firefly luciferase values were normalized to *Renilla* values. Each condition was tested in three independent experiments performed in triplicate.

### Real-time RT-PCR

A total of 43 prostate adenocarcinomas from radical prostatectomies performed for T2 to T3 stage tumors were obtained from the archives of the Department of Pathology, Clinic Hospital of Barcelona. The Gleason sum score ranged from 4 to 9 and were stratified as grade < 7 (7 patients) versus > 7 (26 patients). Prostatic tissue from the benign peripheral zone (BPZ) could be evaluated in 10 specimens. Total RNA was prepared from cells or tissues with RNeasy kits (Qiagen, Valencia, CA) and cDNA synthesis was performed with the High-Capacity cDNA Reverse Transcription Kit (Applied Biosystems, Foster City, CA). Real-time RT-PCR was performed with the Universal Probe Library system (Roche, Mannheim, Germany) on a LightCycler 480 RealTime PCR instrument (Roche). Specific primers used are shown in Supplementary Information Additional file 1: Table S2. Since the relative amplification efficiencies of target and reference samples were found to be approximately equal, the ΔΔCt method was applied to estimate relative transcript levels. RPS14 amplification levels were used as internal references. Data in triplicates were calculated and presented as mean + SEM.

### Immunofluorescence

Immunofluorescence assays of cultured cells were performed as described [16]. DNA was stained with Hoechst 33258. Fluorescent images were captured by confocal microscopy (FV1000, Olympus, Tokyo, Japan) and quantified with Olympus Fluorview software.

### Chromatin immunoprecipitation (ChIP)

Chromatin was immunoprecipitated using EZ-chip Chromatin Immuno Precipitation kit (Millipore, Billerica, MA). Briefly, after a mild formaldehyde crosslinking step, cells were sonicated, lysates incubated with primary antibodies and precipitated with protein A/G-Sepharose. Crosslinking of DNA-protein complexes was reversed, DNA purified and used as a template for PCR reactions. Primers used for PCR in ChIP experiments are described in Additional file 1: Table S3.

### Pull-down assays

GST-fusion proteins expressed in *Escherichia coli* BL-21 strain were purified and stored at -80°C. Pull down assays were performed as described [16]. Protein complexes were analyzed by SDS-PAGE and Western blotting.

### Western blotting

Western blotting was performed as described previously [16]. Reactivity was detected with a chemiluminescent substrate (ECL, Amersham Biosciences).

### *In vitro* invasion assays

Assays were performed using growth factor-reduced Matrigel-coated 8-μm pore size Transwell chambers (BD Bioscience, Franklin Lakes, NJ). Invasive cells at the bottom chamber were stained with Hoechst 33258 and scored. Each condition was tested in triplicate.

### Spheroid formation assays

Cells (10^3^ cells/well) were plated in triplicate samples in 24-well Ultra Low Attachment plates (Corning) in 1 mL of complete medium containing 0.75% methylcellulose (Sigma-Aldrich) and grown for 14 days before counting.

### *In vivo* tumorigenic assays

The firefly luciferase gene was integrated into the genome of PC-3 cells by lentiviral transduction of a pIRE-LTXT-based construct. shControl and PC-3 shPTOV1 cells (3×10^6^ cells/100 μL PBS:Matrigel (1:1)) were subcutaneously implanted in the rear right flank of 6 week-old male SCID-Beige mice (n = 5 for each cell line) (Charles River Laboratories, Barcelona, Spain). All animal experimental procedures were approved by the Vall d’Hebron Hospital Animal Experimentation Ethic Committee. Tumor growth was monitored twice a week by caliper measurements (D × d^2^/2, where D is the major diameter and *d* the minor diameter) and *in vivo* bioluminescence imaging (BLI). BLI intensity was quantified in photons per second (ph/s) using the IVIS Spectrum Imaging System equipped with the Living Image 4.0 software (Caliper Life Sciences). After reaching 1.5 cm in diameter, mice were anesthetized and primary tumors excised, weighted and imaged by *ex vivo* BLI. Tumor/control (T/C) weight ratio was calculated by dividing the median value of the tumor weight of the test tumors by the median value of the control group. Mice were monitored for metastatic growth after tumor excision to detect secondary metastases by *in vivo* bioluminescent imaging using the IVIS Spectrum. Experimental end-point was metastasis detection, after which mice were euthanized and selected tissues analyzed by *ex vivo* BLI and then processed for histopathology.

### Immunohistochemistry

Samples from 20 prostate adenocarcinomas, 10 with Gleason < 7 and 10 with Gleason > 7, plus 16 metastases to regional lymph nodes were obtained from the archives of the Department of Pathology, Hospital Vall d’Hebron and the Clinic Hospital of Barcelona. The study was approved by the Institutional Ethical Boards at the Vall d´Hebron Research Institute and the Clinic Hospital. Four μm consecutive or nearly consecutive sections were analyzed by immunohistochemistry with the avidin-biotin peroxidase method. As a negative control, non-specific rabbit antibody was used and gave clean negative results in all cases tested. Positivity was considered when > 10% of the cells showed unequivocal staining. PTOV1, HEY1 and HES1 expression were evaluated in a semiquantitative manner [6,8], whereby the levels of expression are represented as the percentage of positive cells and the intensity of staining [Hscore = 1 × (% weak) + 2 × (% moderate) + 3 × (% intense) in a range between 0 and 300] [6,8],

### Fly strains and experiments

The Notch alleles *N*^55e11^ and *N*^Ax-M1^ and *nubbin*-*Gal4* and *engrailed-Gal4UAS-GFP* lines were obtained from the Bloomington Stock Center (Indiana University, Bloomington, IN). The generation of the transgenic lines is described in the Supporting Information. For immunohistochemistry, third instar larval discs were dissected, fixed and processed for staining with specific antibodies. Quantification of wing areas was performed using the NIH ImageJ software.

### Statistics

Results are expressed as means + standard errors of the means. The 2-tailed Student’s *t* test was used for statistical analysis. A *p* value < 0.05 was taken as the level of significance. To analyze distributions of qualitative variables, the Pearson coefficient was used. These analyses were performed using the Excel package.

## Abbreviations

PC: Prostate cancer; DAPT: N-[N-(3,5-Difluorophenacetyl)-L-alanyl]-S-phenylglycine t-butyl ester; RA: Retinoic acid; ΔE: Intracellular partially activated Notch1; ICN: Intracellular activated Notch1; MED: Mediator; HGPIN: High grade intraepithelial neoplasia; HDAC: Histone deacetylase; SMRT/NCoR: Silencing mediator of retinoid and thyroid hormone receptors/nuclear receptor co-repressor; T-ALL: T cell acute lymphoblastic leukemia; p300: Histone acetyl-transferase; CBP: Histone acetyl-transferase CREB-binding protein; dnMAML1: Dominant-negative of Mastermind-like gene.

## Competing interest

The authors declare that they have no competing interest.

## Authors’ contributions

Conceived and designed the experiments: R Paciucci. Performed the in vitro culture studies: L Alaña, M Sesé, V Cánovas, C Ruiz. Performed the animal experiments: Y Fernandez, I Abasolo. Supporting technical experiments: Y Puñal, PL Fernandez. Performed the Drosophila experiments: M Sesé, M Corominas, F Serras. Analyzed the data: R Paciucci, L Alaña, M Sesé, I deTorres, T Thomson. Contributed reagents/materials/analysis tools: L Espinosa, A Bigas, S Ramón y Cajal. Wrote the manuscript: R Paciucci, TM Thomson. All authors read and approved the final manuscript.

## Supplementary Material

Additional file 1: Table S1 shRNA sequences used for PTOV1 knockdown. **Table 2SA.** Primers used for real-time RT-PCR using Universal Probe Library (Roche). **SB.** Primers used for real-time RT-PCR using SYBR Green (Life Technology). **Table S3.** Primers used for Chromatin inmunoprecipitation (ChIP). **Figure S1.** The levels of transcription of the Notch target genes *HES1* and *HEY1* in LNCaP prostate cancer cells are modulated by the γ-secretase inhibitor DAPT. **Figure S2.** The four different Notch receptors are expressed at variable levels in human prostate cell lines. **Figure S3.** Western blots illustrating the degree of PTOV1 knockdown in RWPE1, RWPE2 and PC-3 cells by shRNA1397 and shRNA1439. **Figure S4.** PTOV1 represses Notch dependent *HES1* expression in HeLa and COS-7 cells. **Figure S5.** PTOV1 interacts with the Notch co-repressor SMRT. **Figure S6(A)** Occupancy by PTOV1 of the endogenous *HES1* promoter under inhibition of Notch signaling. **S6(B)** Occupancy by the co-repressor NCoR of the endogenous *HEY1* promoter under inhibition of Notch signaling. **Figure S7.** PTOV1 promotes proliferation, anchorage-independent growth and repression of Notch targets genes *HES1* and *HEY1* in HaCaT transformed keratinocytes. Click here for file
