# Supplementary material for: Prostate tumor OVerexpressed-1 (PTOV1) down-regulates HES1 and HEY1 notch targets genes and promotes prostate cancer progression
Source: Mol Cancer. 2014 Mar 31;13:74. doi: 10.1186/1476-4598-13-74 (PMC4021398; doi:10.1186/1476-4598-13-74)
Supplement: Additional file 1: Table S1 — shRNA sequences used for PTOV1 knockdown. Table 2SA. Primers used for real-time RT-PCR using Universal Probe Library (Roche). SB. Primers used for real-time RT-PCR using SYBR Green (Life Technology). Table S3. Primers used for Chromatin inmunoprecipitation (ChIP). Figure S1. The levels of transcription of the Notch target genes HES1 and HEY1 in LNCaP prostate cancer cells are modulated by the γ-secretase inhibitor DAPT. Figure S2. The four different Notch receptors are expressed at variable levels in human prostate cell lines. Figure S3. Western blots illustrating the degree of PTOV1 knockdown in RWPE1, RWPE2 and PC-3 cells by shRNA1397 and shRNA1439. Figure S4. PTOV1 represses Notch dependent HES1 expression in HeLa and COS-7 cells. Figure S5. PTOV1 interacts with the Notch co-repressor SMRT. Figure S6(A) Occupancy by PTOV1 of the endogenous HES1 promoter under inhibition of Notch signaling. S6(B) Occupancy by the co-repressor NCoR of the endogenous HEY1 promoter under inhibition of Notch signaling. Figure S7. PTOV1 promotes proliferation, anchorage-independent growth and repression of Notch targets genes HES1 and HEY1 in HaCaT transformed keratinocytes. [file 1476-4598-13-74-S1.docx]

**Prostate Tumor OVerexpressed-1 down-regulates HES1 and HEY1 Notch targets genes and promotes prostate cancer progression**

Lide Alaña, Marta Sesé, Verónica Cánovas, Yolanda Puñal, Yolanda Fernández, Ibane Abasolo, Inés de Torres, Cristina Ruiz, Lluís Espinosa, Anna Bigas, Santiago Ramón y Cajal, Pedro L. Fernández, Florenci Serras, Montserrat Corominas, Timothy M. Thomson and Rosanna Paciucci

**Contents**:

Supplementary Tables 1-3

Supplementary Figures 1-7 with Legends

**Supplementary Table 1.** shRNA sequences used for PTOV1 knockdown.

| **shRNA** | **ID** | **Sequence** |
| --- | --- | --- |
| PTOV1 (sequence 1439) | Sigma 143905 | CCGGCCTGTACTCTTCAGAGAAGAACTCGAGTTC  TTCTCTGAAGAGTACAGGTTTTTTG |
| PTOV1 (sequence 1397) | Sigma 139737 | CCGGCCTGTACTCGTCCAAGAAGAACTCGAGTT  CTTCTTGGACGAGTACAGGTTTTTTG |
|  | | |

**Supplementary Table 2A.** Primers used for real-time RT-PCR using Universal Probe Library (Roche).

|  | **UPL probe** | **Forward primer** | **Reverse primer** |
| --- | --- | --- | --- |
| **PTOV1** | 9 | 5’-gcttcgtcagtgccatcc-3’ | 5’ tgagttgacaccaccaggtc 3’ |
| **HES1** | 60 | 5’ agtgaagcacctccggaac 3 | 5’-cgttcatgcactcgctga-3’ |
| **HEY1** | 29 | 5’-catacggcaggagggaaag-3’ | 5’-gcatctagtccttcaatgatgct-3’ |
| **RPS14** | 81 | 5’-ggcagagagatgaatcctca-3’ | 5’-caggtccaggggtcttggtc-3’ |

**Supplementary Table 2B.** Primers used for real-time RT-PCR using SYBR Green (Life Technology).

| **NOTCH1** | 5’-GAGCAGATTTTTGCAATACC-3’ | 5’-GCATGACACACAACAGACTC-3’ |
| --- | --- | --- |
| **NOTCH2** | 5’-GTGAACCCTGTAAGAATGGA-3’ | 5’-TCAGTGCACTCATTGATGTT-3’ |
| **NOTCH3** | 5’-AGACGCTCGTCAGTTCTTAG-3’ | 5’-TGGAAAGAGAAGAGGATGAA-3’ |
| **NOTCH4** | 5’-TGTGTAGGTGCTGAAAAGTG-3’ | 5’-TAGCAGTGGCTAGAAGAAGC-3’ |

**Supplementary Table 3.** Primers used for Chromatin inmunoprecipitation (ChIP).

| ***Primer*** | **sequence** |
| --- | --- |
| **HEY1 promoter *forward*** | 5’ TCAGTGTGTGCGGAACGCAAG 3’ |
| **HEY1 promoter *reverse*** | 5’ TTCTTCACCTCGATGGTCTCGTC 3’ |
| **HES1 promoter *forward*** | 5’ GCGTGTCTCCTCCTCCCATT 3’ |
| **HES1 promoter *reverse*** | 5’ CCTGGCGGCCTCTATATATA 3’ |
| **HES1 gene *forward*** | 5’ TACCTCTCTCCTTGGTCCTGGACC 3’ |
| **HES1 gene *reverse*** | 5’ CAGATGCTGTCTTTGGTTTATCCG 3’ |

**Supplementary Figures**

**
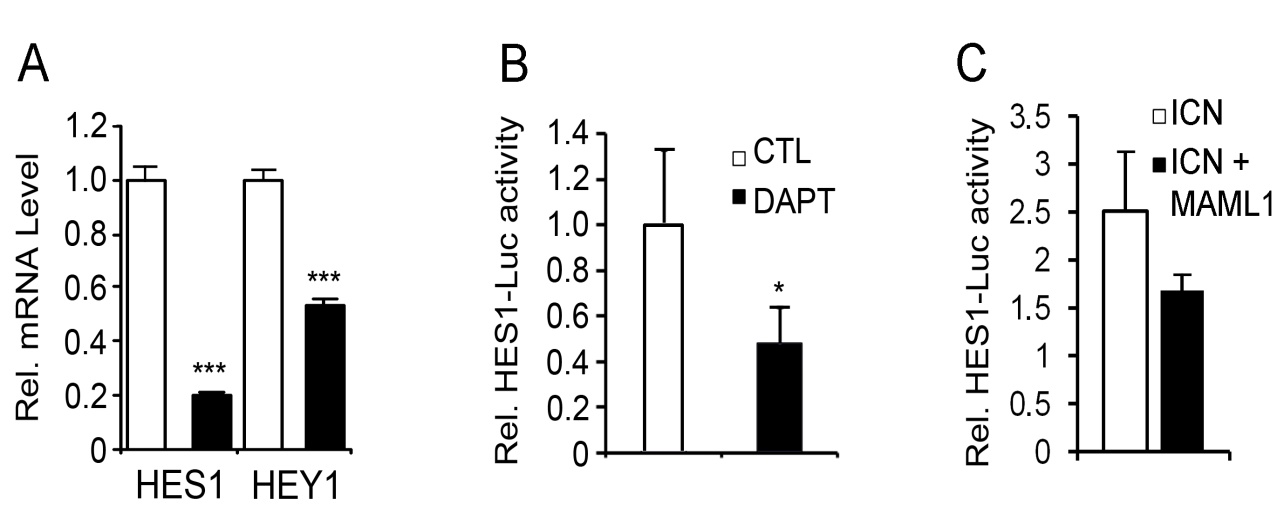
**

**Supplementary Figure 1.** The levels of transcription of the Notch target genes *HES1* and *HEY1* in LNCaP prostate cancer cells are modulated by the γ-secretase inhibitor DAPT. (**A**) Cells were treated with either DAPT or solvent for 4 days and *HES1* or *HEY1* transcript levels quantified by real-time RT-PCR. (**B and C**) The transcriptional activity of the *HES1* promoter is modulated by DAPT and dnMAML1 in LNCaP cells. (**B**) Cells were transfected with *HES-Luciferase* and *TK-Renilla*, treated for 4 days with either DAPT or solvent, and analyzed for luciferase activity. (**C**) Cells were transfected with ICN, *HES-Luciferase* and *TK-Renilla*, cotransfected with either dnMAML1 or pcDNA3 as control, and analyzed for luciferase activity. *Firefly* luciferase values were normalized relative to *Renilla* values.

Statistical significance: * *p* < 0.05, *** *p* < 0.0001.

**
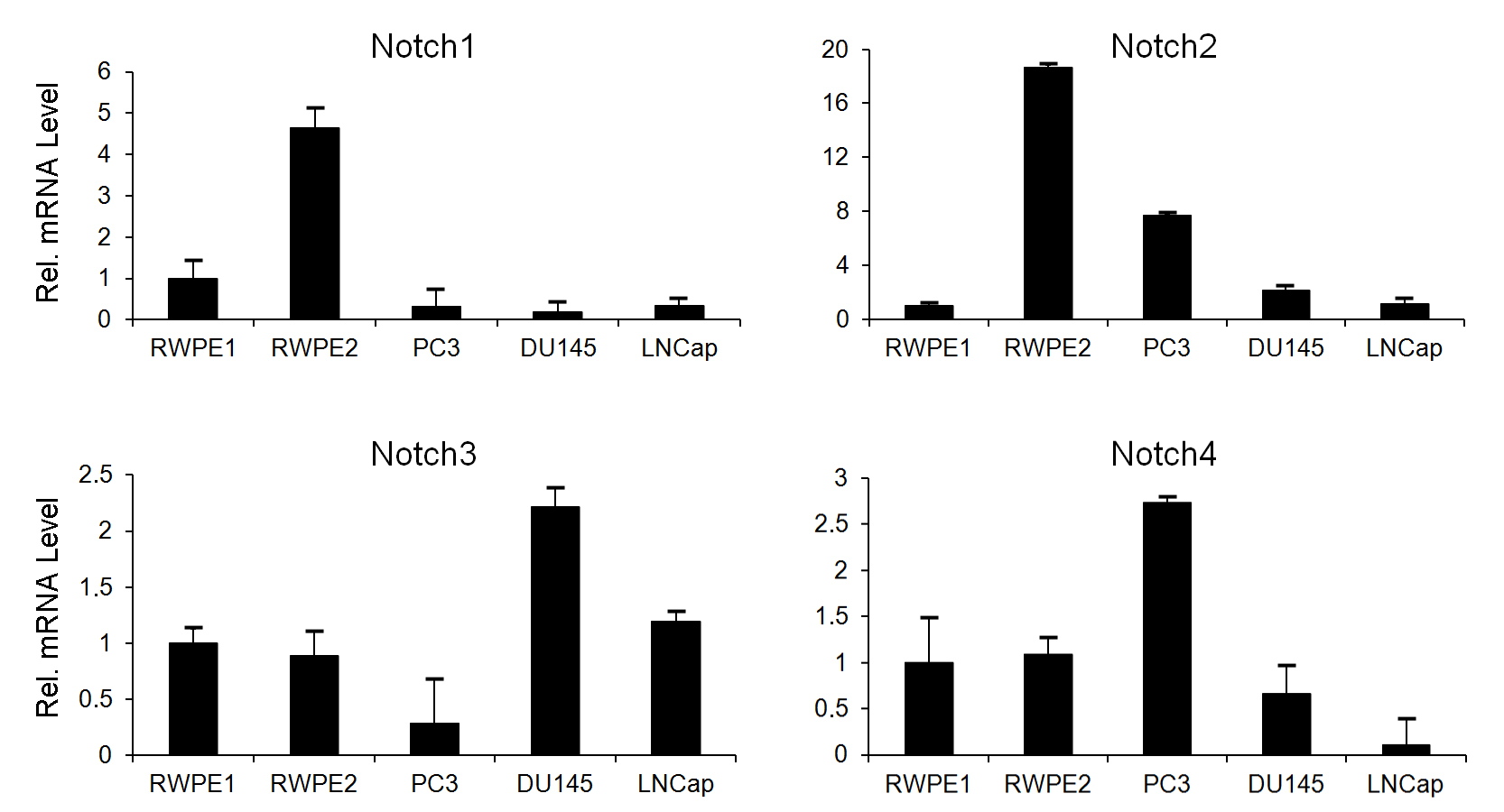
**

**Supplementary Figure 2.** The four different Notch receptors are expressed at variable levels in human prostate cell lines. Normal prostate derived cell lines RWPE1 and RWPE2 and metastasis derived PC-3, DU145 and LNCaP cells were analyzed for the expression of Notch1, Notch2, Notch3 and Notch4 by real-time RT-PCR. Shown are the values normalized to RPS14 and for the relative values in cells RWPE1.

**Supplementary Figure 3.** Western blots illustrating the degree of PTOV1 knockdown in RWPE1, RWPE2 and PC-3 cells by shRNA1397 and shRNA1439. Equivalent amounts (50 μg) of total protein lysates were loaded for each sample. Tubulin signal was used as a control for protein loading. Numbers below the bands indicate the level (percentage) of PTOV1 protein expression relative to tubulin.

**
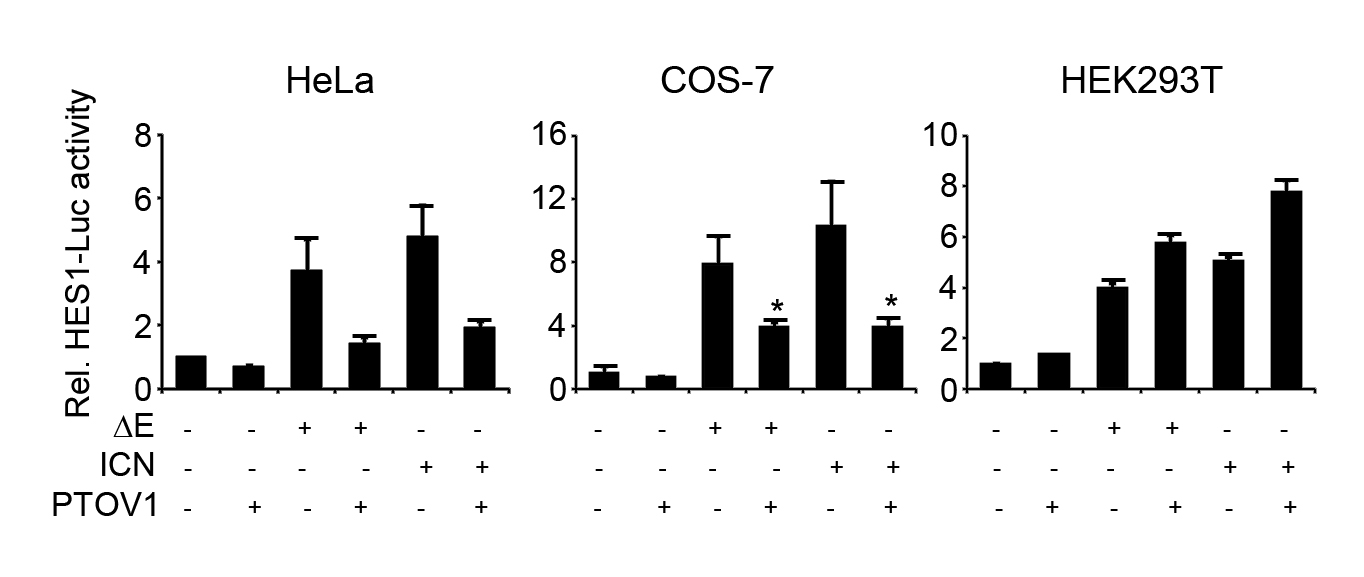
**

**Supplementary Figure 4.** PTOV1 represses Notch dependent *HES1* expression in HeLa and COS-7 cells**.** Exogenous *HES1* promoter activity is negatively regulated by PTOV1 in different cells. HeLa, COS-7, and HEK293T cells were transfected with partially (ΔE) or fully active Notch (ICN) forms, HA-PTOV1 and *HES-Luciferase.* Luciferase activity was analyzed 48 h after transfection and normalized to *Renilla* values. Results shown are from three separate experiments, each performed in triplicate. Statistical significance: * *p* < 0.05.

**
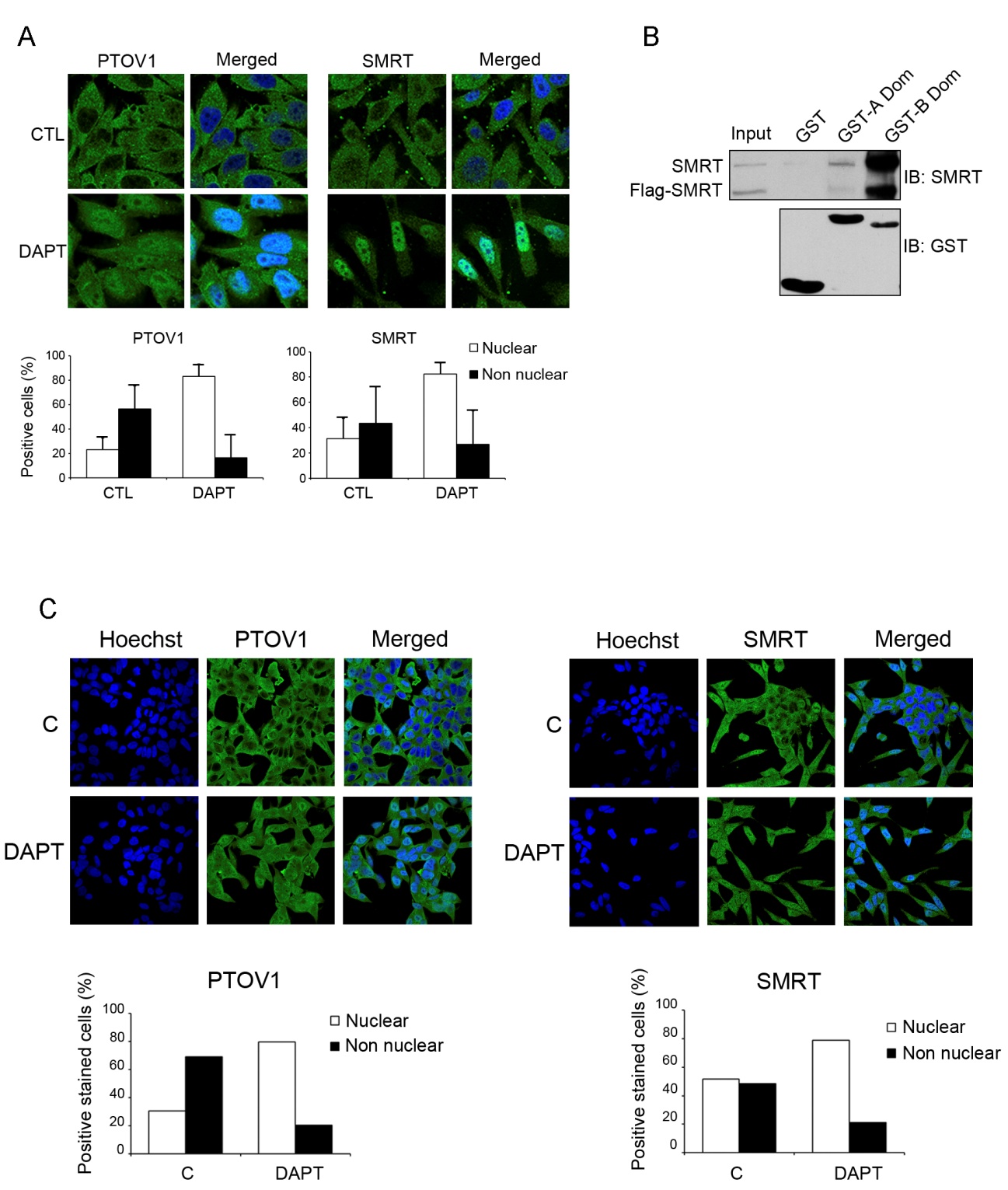
**

**Supplementary Figure** 5. PTOV1 interacts with the Notch co-repressor SMRT. (**A**) PTOV1 and SMRT localize in the nuclei of PC-3 cells treated with DAPT. Cells were treated with DAPT as in Figure 1 and analyzed by immunocytochemistry. Top: Representative images of PC-3 cells. Cells were fixed, permeabilized and stained for endogenous PTOV1 (left panels) or SMRT (right panels). DNA was stained with Hoechst 33258 (blue). Bottom: Cells were scored for nuclear or non-nuclear staining for PTOV1 or SMRT under a fluorescent microscope. At least 300 cells were counted per condition in two independent experiments. (**B**) SMRT interacts *in vitro* with both A and B PTOV1 domains by pull-down assays. GST alone, GST-A domain and GST-B domain fusion proteins bound to Glutathione-Sepharose beads were incubated with extracts from PC-3 cells transfected with Flag-SMRT and bound proteins analyzed by Western blotting with antibodies to SMRT and GST. (**C**) PTOV1 and SMRT co-localize in the nuclei of LNCaP cells treated with DAPT. Representative images of LNCaP cells treated, or not, with DAPT for 4 days. Cells were fixed, permeabilized and stained for endogenous PTOV1 (green, left panel) or SMRT (green, right panel). Nuclear staining was evidenced by counterstaining with Hoechst 33258 (blue). Graph: the number of cells with positive nuclear staining and Non-nuclear staining for PTOV1 or SMRT were scored in each case under a fluorescent microscope. At least 300 cells were scored in each case.

**
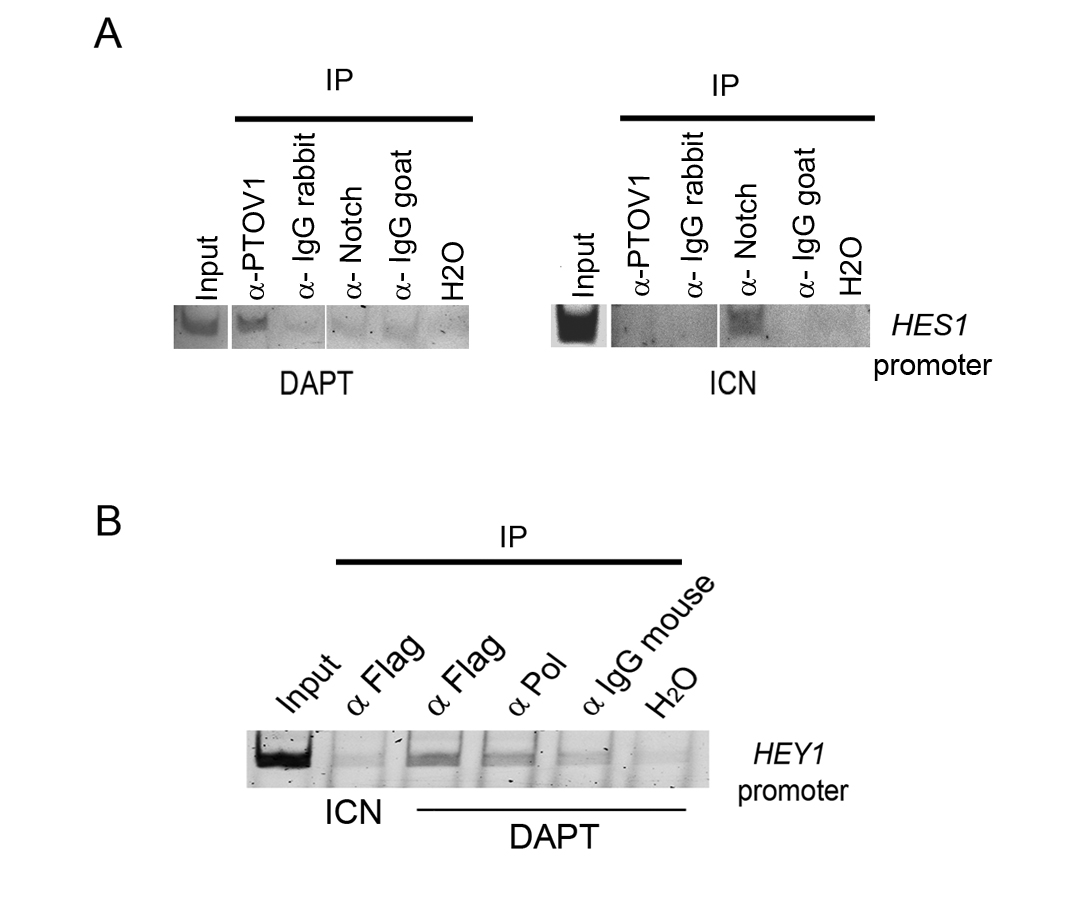
**

**Supplementary Figure 6.** (**A**) Occupancy by PTOV1 of the endogenous *HES1* promoter under inhibition of Notch signaling. PC-3 cells were transfected with FLAG-RBP-Jκ and treated with DAPT or transfected with active Notch1 (ICN), as in Figure 4. Cells were lysed and immunoprecipitated with antibodies to PTOV1, FLAG, Notch or control antibodies. Associated DNA fragments were analyzed by PCR reactions with primers specific for *HES1* promoter regions. (**B**) Occupancy by the co-repressor NCoR of the endogenous *HEY1* promoter under inhibition of Notch signaling. PC-3 cells were transfected with FLAG-NCoR and treated with DAPT or transfected with ICN as above. Immunoprecipitations were performed with the indicated antibodies, and endogenous *HEY1* promoter sequences detected by PCR with specific primers.

**
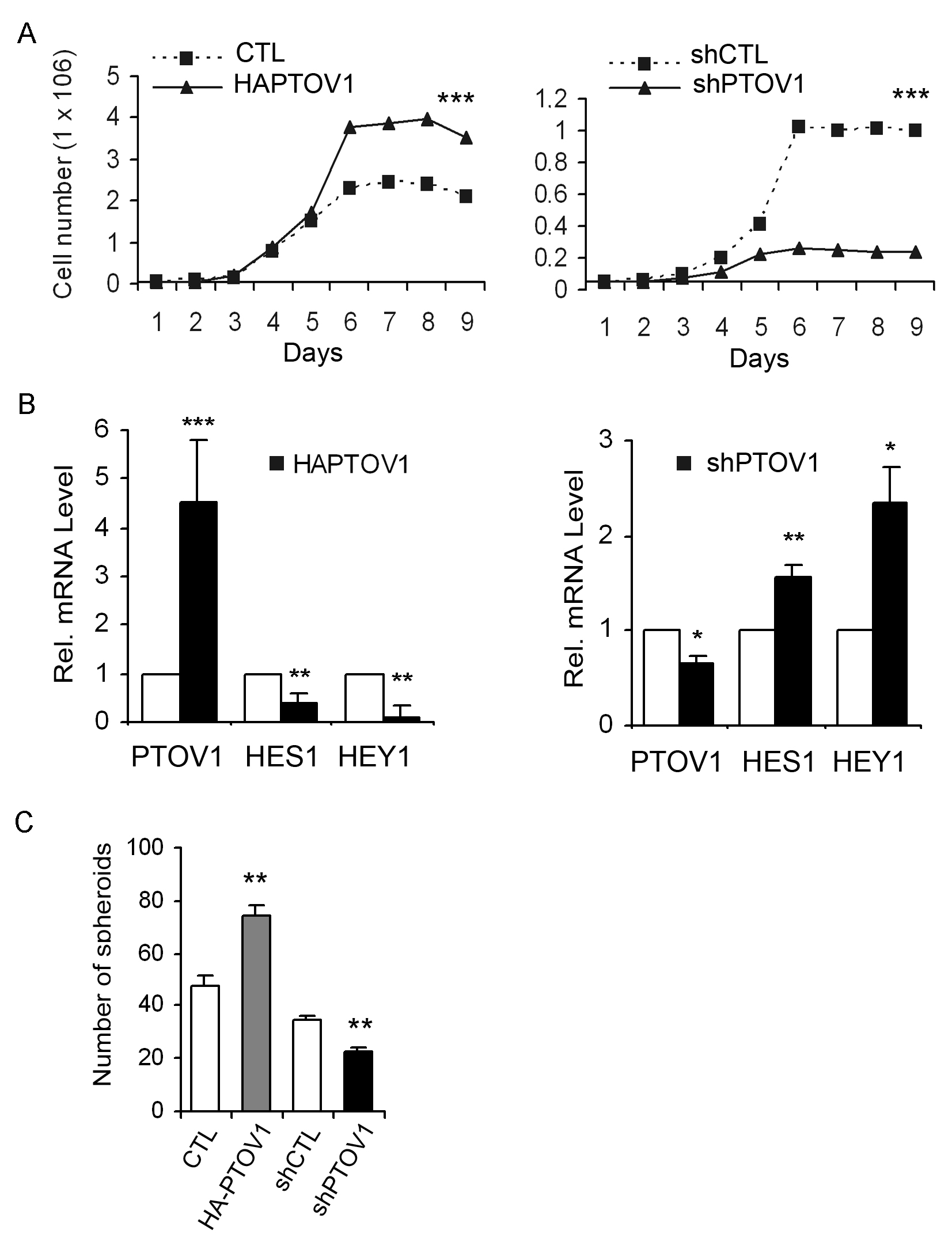
**

**Supplementary Figure 7**. PTOV1 promotes proliferation, anchorage-independent growth and repression of Notch targets genes *HES1* and *HEY1* in HaCaT transformed keratinocytes. Spontaneously transformed HaCaT skin keratinocytes were lentivirally infected either to stably overexpress HA-PTOV1 or to stably knockdown its expression by shPTOV1 1397. (**A**) PTOV1 induces proliferation in HaCaT keratinocytes. Cell proliferation was monitored for 9 days by trypsinization and counting in triplicate assays. (**B**) Modulation of PTOV1 expression levels affects Notch targets expression. Total mRNA was purified from HA-PTOV1 or shPTOV1 infected HaCaT cells and expression of *HES1* and *HEY1* analyzed by real-time PCR. (**C**) PTOV1 promotes anchorage-independent growth of HaCaT keratinocytes. Cells either overexpressing PTOV1 or knocked down with shPTOV1 were seeded in low-attachment plates in the presence of 0.75% methylcellulose and spheroids were counted after 14 days in triplicate assays. Statistical significance: ** *p* < 0.05, ** *p* < 0.001, *** *p* < 0.0001.
